# Anti-Neuroinflammatory Cannabinoid Acids as a New Therapeutic Approach for Multiple Sclerosis

**DOI:** 10.3390/molecules31071227

**Published:** 2026-04-07

**Authors:** Nitsan Sharon, Yvonne Ventura, Nirit Bernstein, Jonathan Gorelick, Shimon Ben-Shabat, Sigal Fleisher-Berkovich

**Affiliations:** 1 Department of Clinical Biochemistry and Pharmacology, Ben-Gurion University of the Negev, Beer-Sheva P.O. Box 653, Israel; kaunitz@post.bgu.ac.il (N.S.); yventura@bgu.ac.il (Y.V.); 2 Institute of Soil Water and Environmental Sciences, Volcani Center, Rishon LeZion 15159, Israel; nirit@volcani.agri.gov.il; 3 Eastern Regional Research and Development Center, Judea Center, Kiryat Arba 90100, Israel; jonathangorelic@gmail.com

**Keywords:** cannabinoid acids, lipopolysaccharide, microglia, neuroinflammation, multiple sclerosis

## Abstract

Neuroinflammation is a hallmark of multiple sclerosis (MS). MS is marked by glial cell activation, autoreactive T cells, and the release of pro-inflammatory cytokines and free radicals. Current therapeutic strategies aim to modulate the immune response using disease-modifying therapies, to slow disease progression. The specific aims of this study were: (a) to investigate the effect of cannabinoid acids on the release of glial neuroinflammatory mediators, (b) to examine the effect of intraperitoneally administered cannabinoid acids on symptoms of MS, and (c) to evaluate their effects on microglial and astrocyte activation and CD4^+^ T cell infiltration into the spinal cords of MS mice. Exposure of BV2 microglia to cannabinoid acids attenuated lipopolysaccharide (LPS)-induced expression of inducible nitric oxide synthase by 40–90% it also reduced the release of nitric oxide and interleukin-17A. Among the cannabinoid acids tested, cannabidiolic acid (CBDA) significantly increased tumor necrosis factor alpha (TNFα) secretion by up to 40% in LPS-stimulated BV2 cells. Intraperitoneal administration of CBDA also resulted in a twofold increase in TNFα secretion in splenocytes isolated from MS mice, compared to untreated MS controls. This study provides evidence that CBDA significantly reduces neurological scores, while both cannabinoid acids attenuate microgliosis, astrogliosis, and CD4+ T cell migration in lumbar spinal cord sections of MS mice. These compounds cross the blood–brain barrier (BBB) and act directly within the central nervous system. The consistent elevation of TNFα in the presence of CBDA across three experimental models suggests a distinctive immunomodulatory role for CBDA, with potential therapeutic implications in MS.

## 1. Introduction

Neuroinflammation is a defining feature of multiple sclerosis (MS), a disease marked by glial activation, autoreactive T cells, and the release of cytokines and free radicals. Demyelination and neuronal loss occur in the context of this inflammatory environment [1,2,3,4]. Current therapeutic approaches focus on modulating the immune response using disease-modifying therapies [5,6,7,8]. Despite this, the pathogenesis of MS is not completely known [9,10,11].

A key contributor to MS pathology is the activation of microglia, the macrophages of the central nervous system (CNS). Microglial activation plays a significant role in both the onset and the progression of MS [12,13]. These cells adopt different activation phenotypes, including classical and alternative activation states. While classical activation causes the release of cytokines, chemokines, and nitric oxide, which contribute to oxidative stress and synaptic impairment, alternative activation supports phagocytosis and tissue regeneration [14].

Tumor necrosis factor-alpha (TNFα) is a key mediator in neuroinflammation and plays a significant role in MS pathogenesis. TNFα is released by dysregulated immune cells and initiates a cascade of inflammatory processes that contribute to demyelination and axonal injury. However, clinical trials using anti-TNFα therapies in MS patients resulted in a more severe, non-remitting disease [15], indicating that TNFα plays a dual role in neuroinflammation, with both pathogenic and protective functions [16].

The migration of CD4^+^ T cells, particularly T helper 1 (Th1) and T helper 17 (Th17) cells, from the peripheral immune system into the CNS is a critical step in MS [17]. These T cells maintain a neuroinflammatory environment characterized by sustained glial activation and demyelination. Th1 cells primarily secrete TNFα, while Th17 cells predominantly produce interleukin-17A [IL-17A; [18]]. IL-17A promotes the release of additional pro-inflammatory cytokines. Microglia including BV2 cells, produce IL-17A under inflammatory conditions. LPS-elevated IL-17A levels [19,20,21,22] have been associated with increased severity of experimental autoimmune encephalomyelitis (EAE), a standard animal model of MS [23].

Cannabinoids, phytochemical compounds found in cannabis plants, modulate the immune response by regulating cytokine production and release, including TNFα levels [24,25]. Cannabinoid acids, including tetrahydrocannabinolic acid (THCA) and cannabidiolic acid (CBDA), are precursors of the main active cannabinoids tetrahydrocannabinol (THC) and cannabidiol (CBD), respectively. CBDA and THCA are the main cannabinoids found in cannabis and have attracted attention for their potential immunomodulatory properties [25]. Structurally, these acidic precursors are distinguished by the presence of a carboxylic acid group at the C-2 position of the aromatic ring, a feature that is lost during decarboxylation into their neutral forms and provides a unique chemical basis for interacting with neuroinflammatory targets [26,27,28].

Notably, activation of peroxisome proliferator-activated receptor gamma (PPARγ), represents a well-established immunomodulatory pathway relevant to MS [29]. A key structural feature shared by virtually all canonical PPAR agonists, both endogenous fatty acids and synthetic ligands, is the presence of an acidic head group, which enables critical hydrogen bonding and electrostatic interactions within the receptor’s ligand-binding domain [30,31]. The carboxylic group of THCA and CBDA may therefore facilitate direct and potentially enhanced engagement of PPAR signaling compared to decarboxylated cannabinoids [32,33]. We now hypothesize that this structural feature confers mechanistically distinct immunomodulatory properties, supporting cannabinoid acids as a differentiated therapeutic strategy in neuroinflammatory disease.

While much of the existing literature focuses on the decarboxylated counterparts THC and CBD, these non-psychoactive acidic precursors offer distinct pharmacological and pharmacokinetic advantages that are particularly relevant to neuroinflammatory pathologies [34]. Both compounds cross the blood–brain barrier (BBB) [35]. In an Alzheimer’s disease (AD)-like model involving neuroinflammation, CBDA and THCA rescued memory deficits and reduced AD- pathology [36,37,38]. Pharmacokinetic studies indicate that CBDA and THCA exhibit greater efficacy and bioavailability than CBD and THC, as reflected by higher peak serum concentrations (C_max_) [39]. Beyond their improved bioavailability, in some cases, these acidic cannabinoids demonstrate greater potency than their neutral counterparts. For example, cannabinoid acids activate PPARγ more effectively than decarboxylated CBD and THC. Phyto cannabinoids that remain poorly characterized or have received limited attention in the literature may offer distinct advantages in modulating neuroinflammation and preventing neurodegeneration. In this study, we investigate for the first time the role of cannabinoid acids in modulating in vitro glial inflammation and EAE mouse model of MS.

## 2. Results and Discussion

**Figure 1:**
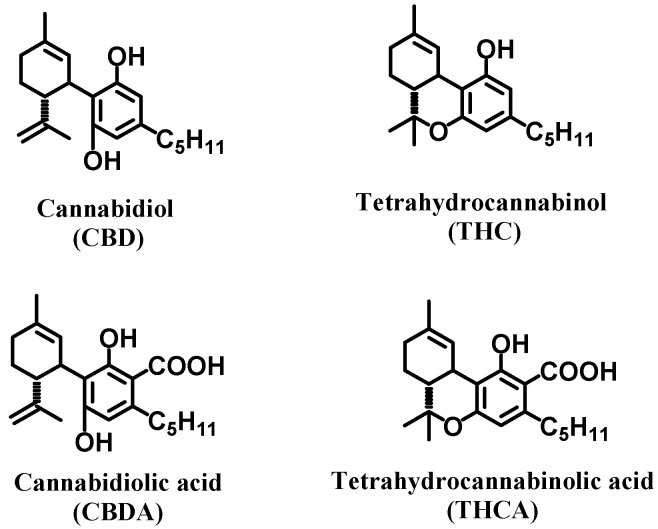
The molecular structures of CBD, THC, CBDA and THCA.

Current research on MS primarily focuses on the mechanisms of neuroinflammation and its associated pathologies, including neurodegeneration. Many studies aim to identify specific subsets of immune cells or signaling molecules involved in the inflammatory cascade and to develop targeted interventions [40,41]. While cytokine production and release are known to play a central role in remodeling the neuroinflammatory environment, the precise mechanisms underlying cytokine regulation during neuroinflammation remain incompletely understood. In this study, we explored the potential of the cannabinoid acids CBDA and THCA (Figure 1) to modulate TNFα and other cytokines implicated in MS pathogenesis. Our findings provide direct evidence supporting immunomodulatory effects of CBDA and THCA in both in vitro and in vivo models, highlighting their potential therapeutic relevance in MS.

**Figure 2:**
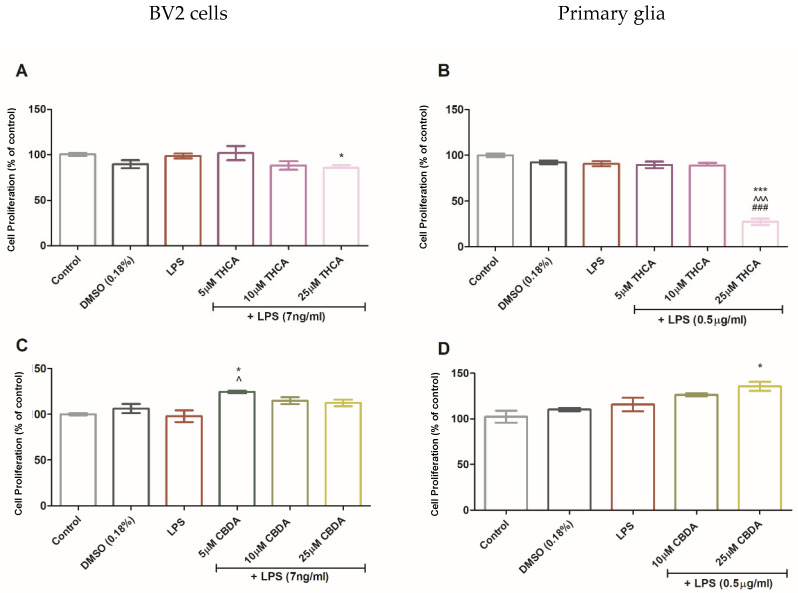
Effect of CBDA and THCA on cell proliferation. BV2 microglial cells (**A**,**C**) and primary mixed glial cultures (**B**,**D**) were incubated for 22 h with LPS (7 ng/mL for BV2 cells or 0.5 μg/mL for primary mixed glia) with or without CBDA or THCA at concentrations of 5, 10, or 25 µM. Cell proliferation was determined using an XTT assay as detailed in Materials and Methods. Data represents results from three independent experiments and are presented as means ± SEM (n ≥ 15). Statistical analysis was performed using one-way ANOVA followed by Tukey–Kramer multiple comparisons post hoc test. * *p* < 0.05 vs. control, *** *p* < 0.001 vs. control, ^ *p* < 0.05 vs. LPS, ^^^ *p* < 0.001 vs. LPS. ### *p* < 0.001 vs. LPS + THC 5 µM.

To assess cytotoxicity, increasing concentrations of THCA (Figure 2A,B) and CBDA (Figure 2C,D) were tested in the presence of LPS in BV2 and rat primary cells. LPS is widely used as a neuroinflammatory stimulus in glial cell models and is known to trigger relapses in EAE mice [42]. Furthermore, elevated LPS levels (endotoxemia) have been described in patients with MS, suggesting a link between peripheral immune activation and CNS pathology [43]. The concentrations for each cannabinoid were selected based on preliminary dose–response screenings also using the XlightlyTT assay. Our objective was to compare the compounds at their respective optimal bioactive ranges rather than on a strictly molar-to-molar basis, since acidic and neutral forms exhibit different pharmacological potencies and uptake kinetics.

THCA did not significantly affect cell viability in BV2 cells, except at 25 µM, which caused a slight reduction. In contrast, 25 µM THCA was toxic to primary glial cultures. Treatment with 5 µM CBDA slightly increased BV2 cell viability relative to the control, and a similar trend was observed in primary cells at 25 µM CBDA.

Our findings show that CBDA and THCA did not exert cytotoxic effects on BV2 microglia in the presence of LPS. This aligns with previous reports, including the study by Nadal et al. which showed neuroprotective effects of THCA via PPARγ signaling.

**Figure 3:**
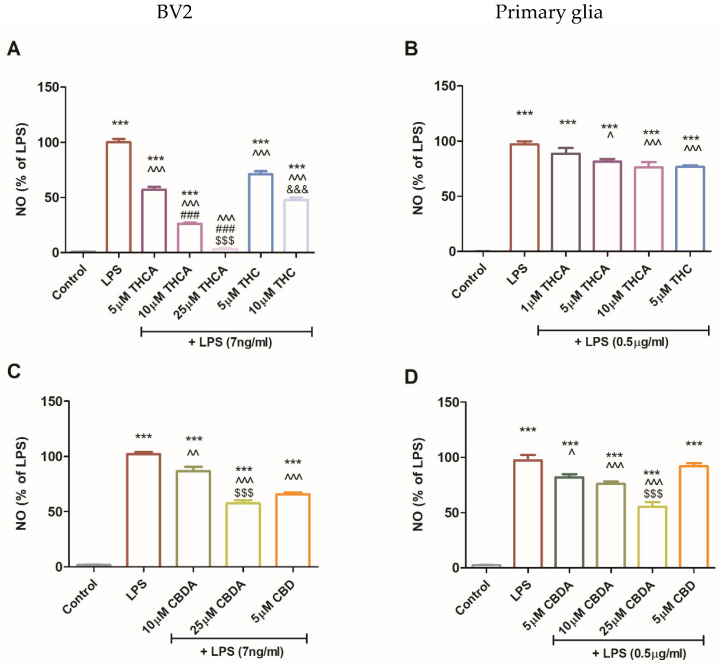
CBDA, THCA, and their neutral derivatives reduce glial NO secretion. BV2 cells (**A**,**C**) and primary mixed glial cultures (**B**,**D**) were incubated for 22 h with LPS (7 ng/mL for BV2 cells; 0.5 µg/mL for primary glia), with or without THCA or THC (**A**,**B**), or CBDA or CBD (**C**,**D**) at different concentrations (5, 10, 25 µM). NO levels in the culture medium were quantified, and the cells were counted at the end of the experiment. Data represents results from three independent experiments and are presented as means ± SEM (n ≥ 15). Statistical analysis was performed using one-way ANOVA followed by Tukey–Kramer multiple comparisons post hoc test. ***—*p* < 0.001 vs. control, ^^^—*p* < 0.001 vs. LPS, ^^—*p* < 0.01 vs. LPS, ^—*p* < 0.05 vs. LPS, ###—*p* < 0.001 vs. LPS + THCA 5 µM, $$$—*p* < 0.001 vs. LPS + CBDA 10 µM, ^&&&^—*p* < 0.001 vs. LPS + THC 5 µM.

Treatment with THCA reduced LPS-induced NO production in a concentration-dependent manner. In BV2 cells, 5 and 25 µM THCA decreased NO levels by 40–95%, while THC at 5 and 10 µM reduced NO secretion by 30 and 50%, respectively (Figure 3A). In LPS-activated primary glial cells, 5 and 10 µM THCA decreased NO production by up to 20%, and a similar effect was observed with 5 µM THC (Figure 3B). Treatment with CBDA at 10 and 25 µM significantly decreased LPS-induced NO secretion in both BV2 cells (Figure 3C) and primary glia (Figure 3D) by 20% and 50%, respectively. Treatment with 5 µM CBD reduced NO production by 40% in BV2 cells (Figure 3C), but this effect was not observed in primary glia (Figure 3D).

These findings suggest that THCA is more effective than THC, suggesting that its acidic form may have enhanced immunomodulatory properties in microglial cells (Figure 3A). In primary mixed glial cultures, both THCA and THC treatments produced comparable reductions in NO secretion. However, treatment with 5 μM CBDA was more effective than CBD in inhibiting NO production in BV2 cells, suggesting distinct mechanisms of action between acidic and neutral cannabinoids and potential cell-type specificity. This cell-type dependence may be attributed to the differential expression of cannabinoid-sensitive receptors on microglia and astrocytes [44,45]. The limited effect of CBD in primary mixed glial cultures, which are predominantly astrocytes, supports this interpretation.

The distinct biological effectiveness of these cannabinoid acids likely stems from their unique structural attributes. Unlike their neutral counterparts, these acidic precursors possess a carboxylic acid functional group (–COOH) at the C-2 position of the aromatic ring, a feature that aligns them structurally with established classes of potent anti-inflammatory agents. Specifically, the presence of a salicylic acid within these molecules creates a structural parallel to classical non-steroidal anti-inflammatory drugs [46]. This acidic group functions as a molecular “anchor”, facilitating a higher density of polar interactions and hydrogen bonding within the binding pockets of target proteins. Interactions that are chemically impossible for neutral cannabinoids to establish. The functional criticality of this free carboxylic group is further validated by the design of synthetic analogs like Ajulemic Acid, which was engineered with an acidic terminus specifically to enhance affinity for the PPARγ receptor while eliminating psychotropic effects [47]. Evidence from prior methylation studies, where masking the acidic group led to a complete loss of Cyclooxygenase-2 selectivity and diminished receptor activation, reinforces the rationale that the acidic form is the primary driver of bioactivity. In addition, the discovery that 7-COOH-CBD acts as a potent anti-inflammatory agent further supports the notion that the presence of a carboxylic acid group is a critical determinant of cannabinoid bioactivity.

**Figure 4:**
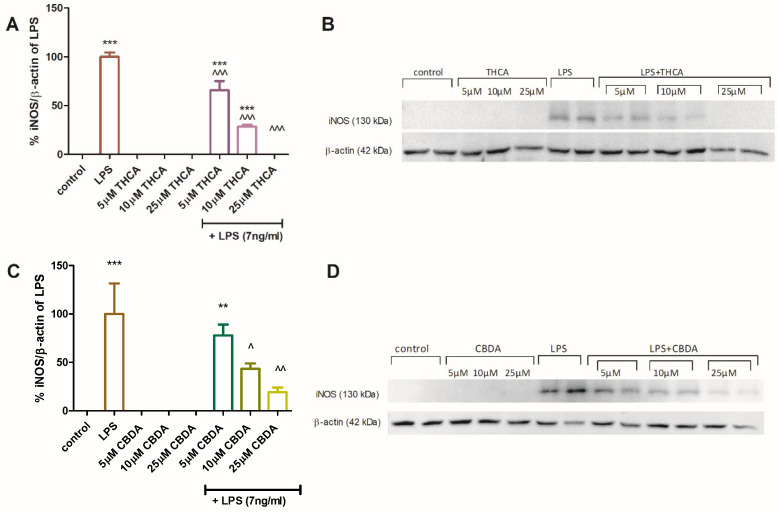
CBDA and THCA reduce iNOS protein expression in BV2 cells. BV2 cells were incubated for 22 h with LPS (7 ng/mL) with or without THCA (**A**,**B**) or CBDA (**C**,**D**) at different concentrations (5, 10, 25 µM). SDS-PAGE and Western blot analysis were performed using antibodies against iNOS and β-actin. The immunoblot images represent two independent experiments. Semi-quantitative analysis (**A**,**C**) was performed using Image J 1.43m software. Data are presented as means ± SEM (n = 4). Statistical significance was determined using one-way ANOVA followed by Tukey–Kramer multiple comparisons post hoc test. *** *p* < 0.001 vs. control, ** *p* < 0.01 vs. control, ^^^ *p* < 0.001 vs. LPS, ^^—*p* < 0.01 vs. LPS. ^—*p* < 0.05 vs. LPS.

The effect of CBDA and THCA on iNOS protein expression in BV2 cells was assessed by Western blot analysis (Figure 4). Semi-quantitative analysis showed that THCA at 5 and 10 µM (Figure 4A,B) and CBDA at 10 and 25µM (Figure 4C,D) significantly reduced iNOS expression in LPS-activated BV2 cells. Neither compound altered iNOS expression in unstimulated cells. These results provide molecular validation for the observed reduction in NO secretion described above. Since iNOS is the primary enzyme responsible for the sustained production of NO during neuroinflammation [48], the downregulation of its protein expression explains the potent inhibitory effects of CBDA and THCA.

**Figure 5:**
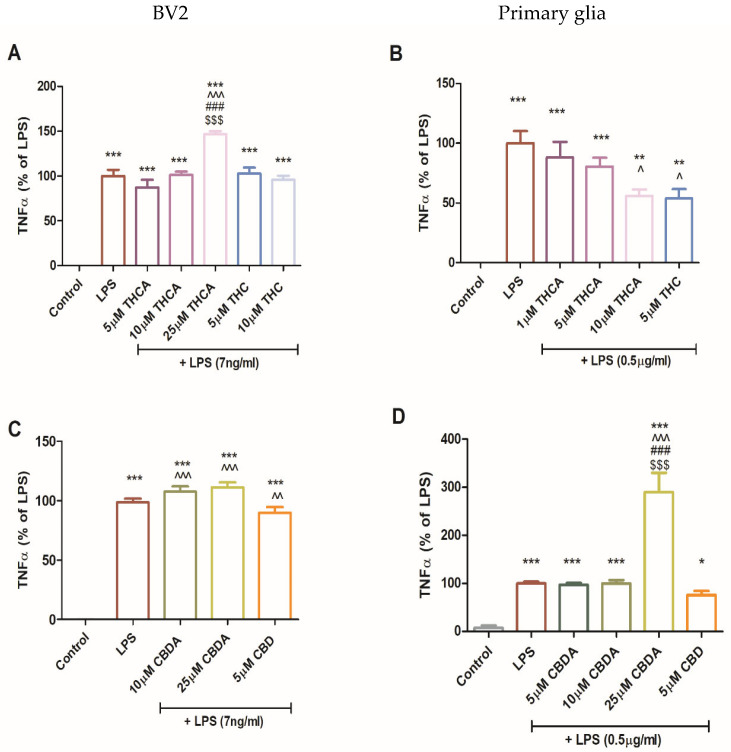
Effect of CBDA and THCA on TNFα production in BV2 and primary glial cells. BV2 (**A**,**C**) and primary glial cells (**B**,**D**) were incubated for 22 h with LPS (7 ng/mL) and treated with CBDA, THCA, or their neutral derivatives (CBD and THC, respectively) at different concentrations (5, 10, 25 µM). Culture media were collected and analyzed for TNFα levels using ELISA. Data represents results from three independent experiments and are presented as means ± SEM (n = 15). Statistical analysis was performed using one-way ANOVA followed by Tukey–Kramer multiple comparisons post hoc test. *** *p* < 0.001 vs. control, ** *p* < 0.01 vs. control, * *p* < 0.05 vs. control, ^^^—*p* < 0.001 vs. LPS, ^^—*p* < 0.01 vs. LPS, ^—*p* < 0.05 vs. LPS*p* < 0.001 vs. LPS + THCA 5 µM, ^$$$^—*p* vs. LPS + THCA 10 µM.

During MS progression, glial-derived pro-inflammatory cytokines contribute to demyelination and sustained neuroinflammation. Previous studies have suggested that modulating glial and T cell responses toward anti-inflammatory cytokine profiles may slow MS progression. In this context, the impact of THCA, CBDA, and their neutral derivatives on TNFα secretion was also examined (Figure 5). In BV2 cells, neither THC nor THCA significantly altered LPS-induced TNFα secretion at most tested concentrations. However, treatment with 25 µM THCA significantly increased TNFα secretion in BV2 cells (Figure 5A). In contrast, in primary mixed glia, treatment with 10 μM THCA and 5 μM THC (Figure 4B) significantly reduced LPS-induced TNFα secretion. Treatment with CBDA at 10 µM and 25 µM in BV2 cells and 25 µM CBDA in primary glia caused a significant increase in TNFα secretion (Figure 5C,D).

While TNFα is widely recognized as a pro-inflammatory cytokine, it also possesses anti-inflammatory and regenerative properties [49,50]. Thus, TNFα plays a dual role in neuroinflammation [16], which may be mediated by two different receptors: TNF receptor 1 (TNFR1) mediates demyelination and apoptosis, while TNF receptor 2 (TNFR2) promotes neuroprotection [51]. In the EAE model, TNFα-deficient mice show delayed disease onset [52,53]. Exogenous TNFα treatment has been shown to reduce disease severity and demyelinating lesions in the brain and spinal cord [54].

The mechanistic basis underlying the CBDA-mediated increase in TNFα remains incompletely resolved. Preservation of cellular metabolic activity in the XTTassay indicates that CBDA does not exert direct cytotoxic effects under the conditions selected (Figure 2). Furthermore, the marked suppression of iNOS expression and NO production (Figure 3 and Figure 4) argues against a mechanism involving nitrosative amplification of TNF-mediated cutotoxicity [55]. However, several limitations prevent definitive attribution of these observations to activation of a protective signaling axis. In particular, the absence of TNFR1/2-specific inhibition studies and the lack of comprehensive downstream pathway analyses, such as evaluation of pro-survival signaling cascades or apoptotic markers, preclude a conclusive mechanistic interpretation.

IL-17A secretion was also measured in LPS-treated BV2 cells. While IL-17A is indeed a hallmark of Th17 lymphocytes, several studies have demonstrated that microglial cells, including the BV2 line, can express and secrete IL-17A under specific inflammatory stimuli, such as LPS [19,20,21,22].

**Figure 6:**
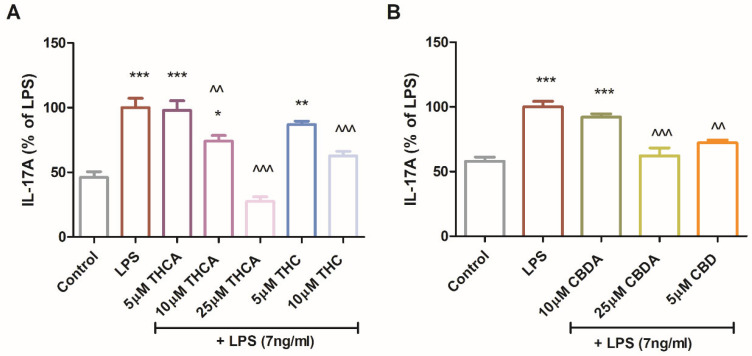
Effect of CBDA, THCA, and their neutral derivatives on IL-17A production in LPS-treated BV2 cells. BV2 cells were incubated for 22 h with LPS (7 ng/mL) and treated with THCA (**A**), CBDA (**B**), or their respective neutral derivatives, CBD and THC, at concentrations of 5 or 10µM Culture media were collected and analyzed for IL-17A levels by ELISA. Data represents results from three independent experiments and is presented as mean ± SEM (n = 15). Statistical analysis was performed using one-way ANOVA followed by Tukey–Kramer multiple comparisons post hoc test. *** *p* < 0.001 vs. control, ** *p* < 0.01 vs. control, * *p* < 0.05 vs. control, ^^^ *p* < 0.001 vs. LPS, ^^ *p* < 0.01 vs. LPS.

THCA reduced IL-17A levels by up to 70% (Figure 6A). CBDA at 25 µM and CBD at 5 µM reduced IL-17A secretion by approximately 30% (Figure 6B). Among the neutral derivatives, 5µM CBD and 10 µM THC decreased IL-17A secretion by 25% and 40%, respectively (Figure 6A,B). CBDA and THCA demonstrated immunomodulatory effects by attenuating LPS-induced expression of iNOS and IL-17A in multiple microglial models. These findings are particularly relevant in the context of neuroinflammation, as both iNOS and IL-17A are closely linked to immune activation and have been implicated in neurodegenerative diseases and brain damage.

**Figure 7:**
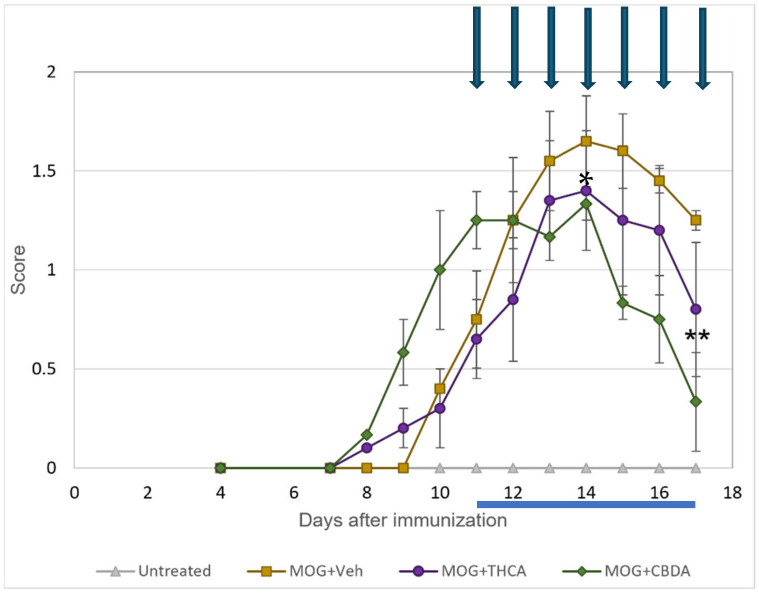
CBDA treatment ameliorates clinical symptoms in MOG-induced EAE mice. Four groups of mice were used: (**a**) MOG-immunized mice (orange); (**b**) MOG-immunized mice treated with THCA (violet); (**c**) MOG-immunized mice treated with CBDA (green) and (**d**) untreated, non-immunized mice (gray). CBDA and THCA were administered intraperitoneally at 10 mg/kg once daily for seven days, starting on day 11 post-immunization (blue arrow). Results are presented as mean ± SEM (n = 4–6). * *p* < 0.05 vs. MOG, ** *p* < 0.01 vs. MOG. Statistical significance was determined using Two-way Repeated-Measures ANOVA followed by Bonferroni post hoc test.

CBDA and THCA were evaluated in vivo using an MOG-induced EAE mouse model. Clinical symptoms onset occurred around day 7 post-immunization (p.i) and progressively worsened over the course of the experiment. Starting on day 11 p.i., mice were treated once daily with CBDA or THCA (10 mg/kg) for seven consecutive days. Both treatments demonstrated a trend toward clinical improvement compared to vehicle-treated MOG-immunized (MOG + Veh) mice. On day 14 and 17 p.i, CBDA-treated mice showed a significantly lower mean clinical score compared to the MOG + Veh group (Figure 7).

These findings suggest that CBDA can attenuate neurological deficits in EAE mice, as evidenced by reduced clinical scores. We acknowledge the small sample size as a limitation of this study. While these cohorts were sufficient to identify significant therapeutic trends, future studies with larger groups are warranted to further validate these findings.

The dissociation between elevated TNFα, reduced other markers and improved clinical outcome argues against a purely pathogenic TNFR1-dominant response. We now explicitly acknowledge the absence of receptor-specific and pathway-level analyses as a limitation and state that future studies will address TNFR1/TNFR2 balance and downstream signaling to mechanistically define CBDA’s immunomodulatory effects.

**Figure 8:**
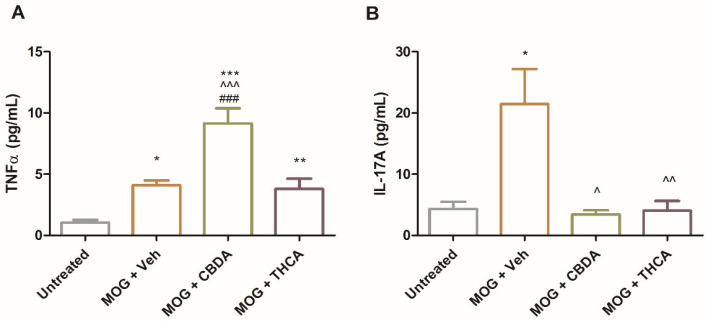
CBDA and THCA modulate TNFα and IL-17A secretion by splenocytes. Splenocytes were isolated from the four experimental groups described in Figure 6, restimulated ex vivo with MOG (1 µg\mL)**,** and cultured for 48 h. Levels of TNFα (**A**) and IL-17A (**B**) in the splenocyte culture supernatants were measured by ELISA. Data are presented as means ± SEM from two independent experiments (n = 12). Statistical analysis was performed using one-way ANOVA followed by Tukey–Kramer multiple comparisons post hoc test *** *p* < 0.001 vs. untreated, ** *p* < 0.01 vs. untreated, * *p* < 0.05 vs. untreated, ^^^ *p* < 0.001 vs. MOG + Veh, ^^ *p* < 0.01 vs. MOG + Veh, ^ *p* < 0.05 vs. MOG + Veh, ### *p* < 0.001 vs. MOG + THCA.

To evaluate the systemic immunomodulatory effects of cannabinoid acids, TNFα and IL-17A secretion were measured in culture media from splenocytes isolated from control mice (untreated), EAE mice (MOG + Veh), or EAE mice treated with MOG and CBDA or THCA. Splenocytes from CBDA-treated EAE mice exhibited a twofold increase in TNFα production compared to the MOG + vehicle group (Figure 8A), whereas no such increase was observed with THCA treatment. In contrast, IL-17A levels were significantly reduced, by over 80%, in splenocyte cultures from both CBDA- and THCA-treated mice relative to the MOG + vehicle group (Figure 8B). These cytokine levels reflect a recall response to MOG stimulation ex vivo.

The consistent elevation of TNFα by CBDA across different models (cell lines, primary cultures, and systemic immune cells) alongside a profound reduction in IL-17A underscores the complex immunomodulatory nature and pleiotropic immunomodulatory effects of CBDA, warranting further investigation into its specific regulatory mechanisms. While the precise biological significance of this divergent response remains to be fully elucidated, the robust inhibition of IL-17A, a key driver of EAE pathology, correlates with the observed clinical improvement.

**Figure 9:**
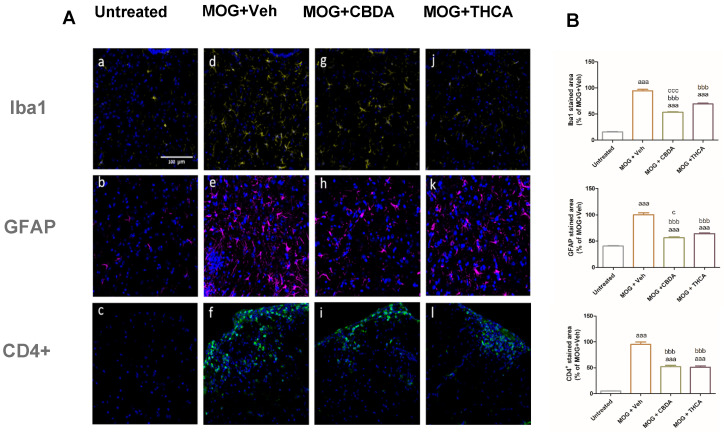
Effect of CBDA or THCA on glial activation and CD4^+^ T cell infiltration in the lumbar spinal cord. (**A**) Representative confocal images of lumbar spinal cord sections stained for microglia (Iba1; panels (**a**,**d**,**g**,**j**)), astrocytes (GFAP; panels (**b**,**e**,**h**,**k**)), and CD4^+^ T cells (CD4; panels (**c**,**f**,**i**,**l**)). Cell nuclei were counterstained with DAPI (blue). Images were acquired at ×40 magnification. Sections were 30 μm thick. The data are representative of two independent experiments; 3–4 sections were analyzed per mouse (n = 6 per group). (**B**) Quantification of Iba-1, GFAP, and CD4 immunoreactivity. Data are presented as means ± SEM and were analyzed using ImageJ software. Statistical analysis was performed using one-way ANOVA followed by Tukey–Kramer multiple comparisons post hoc test. aaa: *p* < 0.001 vs. control, bbb: *p* < 0.001 vs. MOG + Veh, ccc: *p* < 0.001 vs. MOG + THCA.

To examine glial activation, microglial and astrocyte responses were assessed in the lumbar region of the spinal cord (Figure 9A: representative images; Figure 9B: quantification). EAE mice showed a significant increase in microgliosis, indicated by Iba-1 immunoreactivity (Figure 9d), and astrocytosis, indicated by GFAP staining (Figure 9e), compared with untreated controls (Figure 9a,b). Treatment with CBDA or THCA significantly attenuated these responses. Microglial activation was reduced by approximately 50% (Figure 9g,j) and astrocytic activation by approximately 30% (Figure 9h,k), relative to the MOG + vehicle group.

Infiltration of CD4+ T cells into the lumbar spinal cord was also evaluated. EAE mice exhibited increased CD4^+^ immunofluorescence intensity, indicating elevated T cell infiltration (Figure 9f). Treatment with CBDA (Figure 9i) or THCA (Figure 9l) led to a significant reduction in CD4^+^ T cell presence in the spinal cord, compared to the MOG + vehicle group.

This study also suggests that THCA, and to a greater extent CBDA, can decrease microgliosis, astrogliosis, and CD4^+^ T cell migration into lumbar spinal cord (Figure 9). These findings are significant in the context of MS pathogenesis, where the activation and CNS migration of CD4^+^ T cells, particularly T helper 1 (Th1) and T helper 17 (Th17) subsets, play a central role [17]. These T cell populations help sustain a neuroinflammatory environment, characterized by sustained glial activation and neurodegeneration, and are associated with EAE severity. Among these, Th1 CD4^+^ cells are particularly relevant, as their cytokines drive inflammation and contribute to disease progression and symptom worsening [56]. The reduction in CD4^+^ T cell infiltration following treatment with cannabinoid acids suggests an immunomodulatory effect that may help mitigate key drivers of MS pathology.

Our findings are consistent with previous studies demonstrating the therapeutic potential of CBDA and THCA in various in vivo models. Additionally, the concentrations of CBDA and THCA used in this study are consistent with those applied in human cell lines and in vivo reinforcing the translational relevance of our findings. CBDA has been shown to exert anti-hyperalgesic and anti-inflammatory effects in a rodent model of inflammation-related pain [57] and to reduce seizure activity, anxiety, and depressive-like behavior in an acute seizure model in rats [58]. THCA has been reported to alleviate liver fibrosis and inflammation in mice, likely through activation of peroxisome proliferator-activated receptors, and to provide neuroprotection in a mouse model of Huntington’s disease [59,60]. Taken together, these findings support the therapeutic promise of cannabinoid acids in the treatment of MS and related neuroinflammatory conditions.

However, in the present EAE model, while both compounds attenuated spinal cord neuroinflammation, only CBDA treatment achieved a statistically significant reduction in clinical scores. Furthermore, the observed elevation of TNFα by CBDA, though associated with clinical stabilization, requires cautious interpretation. We explicitly acknowledge the absence of TNFR1/2-specific inhibition and downstream signaling analyses as a limitation of this study. Future research is warranted to fully define the TNFR1/TNFR2 balance and mechanistically elucidate the neuroprotective potential of these non-psychoactive cannabinoid acids.

## 3. Materials and Methods

The agents used included CBDA, THCA, and lipopolysaccharides (LPS) from *E. coli* Serotype 055: B5 (Sigma-Aldrich Israel Ltd., Rehovot, Israel). CBDA and THCA were isolated from plant material and analytically characterized prior to biological testing. Analytical HPLC purity was determined using a reversed-phase C18 column with UV detection. Both compounds showed chromatographic purity ≥95%. Identity was further confirmed by LC–MS analysis based on the expected molecular ions and fragmentation patterns. Representative chromatograms and spectra are provided in the Supporting Information (Figures S1 and S2). To minimize decarboxylation, both compounds were stored as dry powders at −20 °C, protected from light and moisture, and prepared fresh in solvent immediately prior to use.

### 3.1. Cell Cultures

#### 3.1.1. BV2 Microglia Cell Cultures

The murine BV2 microglial cell line (see Acknowledgments for line details) was grown in RPMI-1640 supplemented with 4 mM L-glutamine, 10% fetal calf serum (FCS), 100 U/mL penicillin, and 100 µg/mL streptomycin. Cells were maintained in a humidified incubator at 37 °C with 5% CO_2_. For experiments, cells were seeded on 24-well plates at a density of 3 × 10^5^ cells per well or 6-well plates at 1 × 10^6^ cells per well and incubated overnight. The following morning, prior to each experiment, the cells were incubated in serum-free medium (SFM) for 4 h. After SFM removal, test agents were added in SFM containing 0.1% bovine serum albumin (BSA), 1% FCS, and 10 mM HEPES buffer, pH 7.4, for different time intervals. BV2 cells were provided by Dr. Rosario Donato, Department of Experimental Medicine and Biochemical Sciences, Section of Anatomy, University of Perugia, Perugia, Italy.

#### 3.1.2. Primary Glial Cell Cultures

Primary neonatal mixed rat glial cell cultures were prepared from whole brains of 1-day-old Wistar rats, according to previously established work [61]. Immunocytochemistry studies, conducted as previously described [61], revealed that these cultures are composed of approximately 80% astrocytes and 20% microglia.

### 3.2. Cell Proliferation

Cells were seeded at a density of 1 × 10^4^ cells per well in 96-well plates and incubated overnight in complete RPMI-1640 medium. The following day, cells were treated with test agents as described above. The XTT assay was used to assess cell proliferation and metabolic activity, based on the reduction of XTT reagent (2,3-Bis-(2-Methoxy-4-Nitro-5-Sulfophenyl)-2H-Tetrazolium-5-Carboxanilide) to an orange water-soluble formazan product by mitochondrial dehydrogenases in metabolically active cells. XTT reagent was prepared by mixing it with the activation reagent (N-methyl dibenzopyrazine methyl sulfate), at a 50:1 ratio, according to the manufacturer’s protocol (Biological Industries, Kibbutz Beit-Haemek, Israel), and added to each well in a 1:2 ratio. After a 1 h incubation at 37 °C, absorbance was measured at 450 nm with a reference wavelength of 650 nm using a microplate reader (Model 680, Bio-Rad, Hercules, CA, USA).

### 3.3. Quantification of NO Levels (Griess Reaction)

NO levels in the media were determined by measuring nitrite levels in the culture supernatants using the Griess reaction. Equal volumes of culture supernatant and Griess reagent (Sigma-Aldrich Israel Ltd., Rehovot, Israel) were mixed in a 96-well plate and incubated in the dark for 15 min at room temperature. Absorbance was measured at 540 nm using a microplate reader (Bio-Rad Laboratories Ltd., Rishon Le Zion, Israel). Nitrite concentrations were calculated based on a sodium nitrite standard curve.

Following each experiment, cells were collected after a 1 h incubation with 1 mL Isoflow sheath fluid (Beckman Coulter, Inc., Brea, CA, USA) at 4 °C and counted using a Countess 3 automatic cell counter (Thermo Fisher Scientific, Waltham, MA, USA).

### 3.4. Quantification of TNFα Levels (ELISA)

TNFα levels in media were determined using the ELISA MAX™ standard set mouse enzyme-linked immunosorbent assay kit (Biolegend, San Diego, CA, USA) according to the manufacturer’s protocol.

### 3.5. SDS-PAGE and Western Blot Analysis

Whole-cell lysates containing 40 µg of protein were separated on 7.5% SDS-polyacrylamide gels and transferred to nitrocellulose membranes. Membranes were blocked with 4% BSA for 90 min at room temperature and incubated overnight at 4 °C with a rabbit anti-iNOS antibody (1:500, Cayman Chemicals, Ann Arbor, MI, USA). After washing, membranes were incubated for 90 min at room temperature with a donkey anti-rabbit secondary antibody conjugated to horseradish peroxidase (1:10,000, GE Healthcare, Buckinghamshire, UK). Immunoreactivity bands were detected using an enhanced chemiluminescence (ECL) solution and visualized with a luminescent image analyzer (Image Quant LAS 500; Cytiva, Uppsala, Sweden). Semi-quantitative analysis was performed using Image J software (version 1.43 NIH, Bethesda, MD, USA). Band intensities were normalized to β-actin protein levels, determined using a mouse monoclonal anti-β-actin-peroxidase antibody (1:20,000; Sigma-Aldrich Israel Ltd., Rehovot, Israel).

### 3.6. Active MOG-Induced EAE Model

Eight-week-old female C57BL/6 mice (Envigo, Jerusalem, Israel) were immunized with myelin oligodendrocyte glycoprotein (MOG) [peptide 35–55] (AnaSpec, Fremont, CA, USA). Each mouse was injected subcutaneously (s.c.) at two sites on the back, adjacent to each of the hind limbs (total volume 200 μL), containing 200 μg MOG emulsified with complete Freund’s adjuvant (BD Biosciences, Sparks, MD, USA) supplemented with 200 μg/mL heat-killed *Mycobacterium tuberculosis* H37RA. Immediately afterward, mice were injected intraperitoneally (i.p.) with 400 ng/mL reconstituted pertussis toxin (Cayman Chemicals, Ann Arbor, MI, USA), with a second dose given two days later. All procedures were conducted in accordance with national and institutional guidelines for the care and use of laboratory animals. After immunization, the mice were evaluated for neurological scores using the following clinical scoring scale: 0 no symptoms; 0.5 tip of tail limp; 1.0 complete tail limpness; 1.5 limp tail and hind leg inhibition; 2.0 limp tail and weakness of hind legs; 2.5 limp tail and hind dragging legs; 3.0 limp tail and partial paralysis of hind legs; 3.5 complete paralysis of hind limbs; 4.0 complete hind leg and partial front leg paralysis; 4.5 complete hind and front leg paralysis; 5.0 moribund. We confirm that animals were randomized into treatment groups at the onset of symptoms. This randomization ensured that both the MOG + Veh and MOG + CBDA groups started with balanced baseline disease severity, confirming that the significant improvements are strictly attributable to CBDA administration (61).

Starting on day 11 post-immunization, mice were treated i.p. once daily for seven consecutive days. Control EAE mice (n = 4) received the vehicle (Veh) solution composed (Tween-20: ethanol:saline 1:1:8). Treatment groups received either CBDA or THCA (10 mg/kg/day) dissolved in the vehicle solution (n = 6 for each treatment). The experiment was terminated 18 days after disease onset. Spleens were harvested under sterile conditions and immediately placed in cold PBS. Cardiac perfusion was then performed using PBS, followed by 4% Paraformaldehyde (PFA).

### 3.7. Splenocyte Cultures

Each spleen was washed with sterile PBS solution containing 1 mM EDTA. The tissue was then mechanically dissociated by pressing it through a 70 µm cell strainer using the back of a syringe piston, in PBS/EDTA solution. The resulting suspension was filtered again into a clean test tube, and cells were pelleted at 300× *g* for 10 min at 4 °C. After discarding the supernatant, cells were treated with red blood cell lysis buffer (150 µM NH_4_Cl, 10 µM NaHCO_3,_ and 1 µM EDTA in DDW) for 10 min at room temperature. Subsequently, PBS/EDTA buffer was added, and cells were centrifuged at 300× *g* for 10 min at 4 °C. After discarding the supernatant, splenocytes were diluted in DMEM culturing medium supplemented with 10% FCS, 100 U/mL penicillin, 100 mg/mL streptomycin, 1% sodium pyruvate, 10 mM HEPES buffer, 1% non-essential amino acids, and 0.02% β-mercaptoethanol. Cells were counted with a Countess 3 automatic cell counter (Thermo Fisher Scientific, Waltham, MA, USA) using Trypan-Blue. Splenocytes were seeded in U-bottom 96-well plates (1 × 10^6^ cells per well). To stimulate the cells, 100 µL of MOG peptide diluted in culture medium to a final concentration of 1 µg/mL was added to each well. Cultures were maintained at 37 °C in a 5% CO_2_ humidified incubator. After 48 h, the medium was collected and cytokine levels were measured using ELISA kits (Biolegend, San Diego, CA, USA) according to the manufacturer’s instructions.

### 3.8. Immunohistochemistry

Spines were fixed in 4% PFA at 4 °C overnight and subsequently incubated in 20% sucrose for 48 h at 4 °C. Spinal cords were subsequently dissected, separated into thoracic and lumbar sections, mounted in OCT (Scigen Scientific, Gardena, CA, USA), snap frozen at −40 °C, and stored at −80 °C. Frozen tissues were sectioned horizontally into 30 µm slices using a microtome cryostat (Leica Biosystems, Vienna, Austria) and stored in ethylene glycol:glycerol:PBS (1:1:2) at −20 °C until further processing.

Lumbar spinal cord sections were washed with PBS supplemented with 0.05% Tween 20 (PBST) and blocked using 1% BSA and 10% host serum (matching the species of the secondary antibody) diluted in PBS with 0.3% Triton X-100. Sections were incubated overnight at 4 °C with the following primary antibodies: mouse anti-glial fibrillary acidic protein (GFAP; 1:400, Millipore), rabbit anti-Iba-1 antibody (1:1000, FUJIFILM Wako), and rat anti-CD4^+^ antibody (1:50, BD Pharmingen). The next morning, sections were washed with PBST and incubated for 1 h at room temperature in a dark environment with the corresponding secondary antibodies: Alexa Fluor 647-conjugated donkey anti-mouse IgG, Alexa Fluor 647-conjugated donkey anti-rabbit IgG, and Alexa Fluor 488-conjugated goat anti rat IgG (1:200, Jackson Immuno Research Inc., West Grove, PA, USA). Secondary antibodies were diluted in 1% BSA and 1% host serum in PBS with 0.15% Triton X-100. Nuclear staining was performed using DAPI (1 µg/mL, Sigma-Aldrich, Rehovot, Israel), followed by additional washes. Sections were then mounted onto glass slides using Immu-Mount (Epredia Netherlands B.V., Breda, the Netherlands) for fluorescence microscopy.

### 3.9. Confocal Imaging Analysis

Quantification analysis of DAPI, Iba-1, GFAP, and CD4^+^ cells in the spinal cords was performed using the appropriate wavelengths (DAPI at 405 nm, Iba-1 and GFAP at 647 nm, CD4^+^ at 488 nm). All images were obtained using an Olympus FluoView FV1000 confocal microscope (Olympus, Hamburg, Germany). The images were analyzed using Image J software (version 1.43 NIH, Bethesda, MD, USA).

## 4. Statistical Analysis

Results are presented as the mean ± SEM for each experiment. To assess the statistical significance of differences between treatment groups, one-way analysis of variance (ANOVA) was performed, followed by a Post Hoc multiple comparison test (Tukey–Kramer Multiple Comparison Test). Unpaired, two-tailed T-test was used for comparison between two treatment groups, and *p* < 0.05 was considered statistically significant. EAE clinical scores were analyzed using a Two-way Repeated-Measures ANOVA, treating ‘Time’ and ‘Treatment’ as the main factors. Post hoc comparisons between groups at individual time points were performed using the Bonferroni test, and *p* < 0.05 was considered statistically significant.

## Data Availability

The datasets generated during and/or analysed during the current study are available from Prof. Sigal Fleisher-Berkovich on reasonable request.

## Supplementary Materials

The following supporting information can be downloaded at: https://www.mdpi.com/article/10.3390/molecules31071227/s1. Figure S1. Representative chromatographic and spectral characterization of CBDA. Figure S2. Representative chromatographic and spectral characterization of THCA.

## Abbreviations

The following abbreviations are used in this manuscript:

MS	Multiple Sclerosis
LPS	Lipopolysaccharide
CBDA	Cannabidiolic Acid (CBDA)
TNF	Tumor Necrosis Factor
BBB	Blood Brain Barrier
PPAR	Peroxisome Proliferator Activator
Th	T helper
IL-17A	Interleukin-17A
EAE	Experimental Autoimmune Encephalomyelitis
THCA	Tetrahydrocannabinolic Acid
NO	Nitric Oxide
CBD	Cannabidiol
THC	Tetrahydrocannabidiol
iNOS	Inducible Nitric Oxide Synthase
TNFR	Tumor Necrosis Factor Receptor
MOG	Myelin Oligodendrocyte Glycoprotein
BSA	Bovine Serum Albumin
ELISA	Enzyme Linked Immunosorbent Assay
ECL	Enhanced Chemiluminescence
Iba	Ionized Calcium binding adaptor
GFAP	Glial Fibrillary Acidic Protein
EDTA	Ethylenediaminetetraacetic Acid
PFA	Perfluoroalkoxy Alkane

## References

[1] Trapp B.D., Peterson J., Ransohoff R.M., Rudick R., Mörk S., Bö L. (1998). Axonal transection in the lesions of multiple sclerosis. N. Engl. J. Med..

[2] Stadelmann C., Wegner C., Brück W. (2011). Inflammation, demyelination, and degeneration—Recent insights from MS pathology. Biochim. Biophys. Acta.

[3] Friese M.A., Schattling B., Fugger L. (2014). Mechanisms of neurodegeneration and axonal dysfunction in multiple sclerosis. Nat. Rev. Neurol..

[4] García-Domínguez M. (2025). Neuroinflammation: Mechanisms, Dual Roles, and Therapeutic Strategies in Neurological Disorders. Curr. Issues Mol. Biol..

[5] Frischer J.M., Bramow S., Dal-Bianco A., Lucchinetti C.F., Rauschka H., Schmidbauer M., Laursen H., Sorensen P.S., Lassmann H. (2009). The relation between inflammation and neurodegeneration in multiple sclerosis brains. Brain.

[6] Frohman E.M., Racke M.K., Raine C.S. (2006). Multiple sclerosis—The plaque and its pathogenesis. N. Engl. J. Med..

[7] Morrow S.A., Clift F., Devonshire V., Lapointe E., Schneider R., Stefanelli M., Vosoughi R. (2022). Use of natalizumab in persons with multiple sclerosis: 2022 update. Mult. Scler. Relat. Disord..

[8] Yamout B., Al-Jumah M., Sahraian M.A., Almalik Y., Khaburi J.A., Shalaby N., Aljarallah S., Bohlega S., Dahdaleh M., Almahdawi A. (2024). Consensus recommendations for diagnosis and treatment of Multiple Sclerosis: 2023 revision of the MENACTRIMS guidelines. Mult. Scler. Relat. Disord..

[9] Boutitah-Benyaich I., Eixarch H., Villacieros-Álvarez J., Hervera A., Cobo-Calvo Á., Montalban X., Espejo C. (2025). Multiple sclerosis: Molecular pathogenesis and therapeutic intervention. Signal Transduct. Target. Ther..

[10] Nylander A., Hafler D.A. (2012). Multiple sclerosis. J. Clin. Investig..

[11] Rahmanzadeh R., Bruck W., Minagar A., Sahraian M.A. (2018). Multiple sclerosis pathogenesis: Missing pieces of an old puzzle. Rev. Neurosci..

[12] Kwon H.S., Koh S.H. (2020). Neuroinflammation in neurodegenerative disorders: The roles of microglia and astrocytes. Transl. Neurodegener..

[13] Plastini M.J., Desu H.L., Brambilla R. (2020). Dynamic Responses of Microglia in Animal Models of Multiple Sclerosis. Front. Cell Neurosci..

[14] Wei H.X., Guan Y.N., Chen P.P., Rao Z.Z., Yang J.S. (2023). Upregulation of EphA4 deteriorate brain damage by shifting microglia M1-polarization via NF-κB signaling after focal cerebral ischemia in rats. Heliyon.

[15] Wiendl H., Neuhaus O., Kappos L., Hohlfeld R. (2000). Multiple sclerosis. Current review of failed and discontinued clinical trials of drug treatment. Nervenarzt.

[16] Zheng C., Zhou X.W., Wang J.Z. (2016). The dual roles of cytokines in Alzheimer’s disease: Update on interleukins, TNF-α, TGF-β and IFN-γ. Transl. Neurodegener..

[17] Kaskow B.J., Baecher-Allan C. (2018). Effector T Cells in Multiple Sclerosis. Cold Spring Harb. Perspect. Med..

[18] Prajeeth C.K., Lohr K., Floess S., Zimmermann J., Ulrich R., Gudi V., Beineke A., Baumgartner W., Muller M., Huehn J. (2014). Effector molecules released by Th1 but not Th17 cells drive an M1 response in microglia. Brain Behav. Immun..

[19] Di Filippo M., Mancini A., Bellingacci L., Gaetani L., Mazzocchetti P., Zelante T., La Barbera L., De Luca A., Tantucci M., Tozzi A. (2021). Interleukin-17 affects synaptic plasticity and cognition in an experimental model of multiple sclerosis. Cell Rep..

[20] Kawanokuchi J., Shimizu K., Nitta A., Yamada K., Mizuno T., Takeuchi H., Suzumura A. (2008). Production and functions of IL-17 in microglia. J. Neuroimmunol..

[21] Li H., Xie F., Cui X., Shen G. (2026). Unraveling the role of IL-17 signaling pathway in breast cancer-related depression: Insights from in vivo/in vitro models and transcriptomic analysis. Funct. Integr. Genom..

[22] Yin J., Li W., Shen L.-P., Zhang W.-L., Chen J.-Y., Zhang B.-B., Chen Y.-J., Li T., Li H.-Z., Gao Z. (2026). Cerebellar microglia-derived IL-17A mitigates autism-related behavioral and synaptic deficits. Mol. Psychiatry.

[23] Constantinescu C.S., Farooqi N., O’Brien K., Gran B. (2011). Experimental autoimmune encephalomyelitis (EAE) as a model for multiple sclerosis (MS). Br. J. Pharmacol..

[24] Hanus L.O., Meyer S.M., Munoz E., Taglialatela-Scafati O., Appendino G. (2016). Phytocannabinoids: A unified critical inventory. Nat. Prod. Rep..

[25] Henshaw F.R., Dewsbury L.S., Lim C.K., Steiner G.Z. (2021). The Effects of Cannabinoids on Pro- and Anti-Inflammatory Cytokines: A Systematic Review of In Vivo Studies. Cannabis Cannabinoid Res..

[26] Tahir M.N., Raz F.S., Rondeau-Gagné S., Trant J.F. (2021). The biosynthesis of the cannabinoids. J. Cannabis Res..

[27] Stone N.L., Murphy A.J., England T.J., O’Sullivan S.E. (2020). A systematic review of minor phytocannabinoids with promising neuroprotective potential. Br. J. Pharmacol..

[28] Takeda S., Misawa K., Yamamoto I., Watanabe K. (2008). Cannabidiolic acid as a selective cyclooxygenase-2 inhibitory component in cannabis. Drug Metab. Dispos..

[29] Drew P.D., Xu J., Racke M.K. (2008). PPAR-gamma: Therapeutic Potential for Multiple Sclerosis. PPAR Res..

[30] Egawa D., Itoh T., Yamamoto K. (2015). Characterization of Covalent Bond Formation between PPARγ and Oxo-Fatty Acids. Bioconjugate Chem..

[31] Miyachi H. (2023). Structural Biology Inspired Development of a Series of Human Peroxisome Proliferator-Activated Receptor Gamma (PPARγ) Ligands: From Agonist to Antagonist. Int. J. Mol. Sci..

[32] D’Aniello E., Fellous T., Iannotti F.A., Gentile A., Allara M., Balestrieri F., Gray R., Amodeo P., Vitale R.M., Di Marzo V. (2019). Identification and characterization of phytocannabinoids as novel dual PPARalpha/gamma agonists by a computational and in vitro experimental approach. Biochim. Biophys. Acta Gen. Subj..

[33] Nadal X., Del Rio C., Casano S., Palomares B., Ferreiro-Vera C., Navarrete C., Sanchez-Carnerero C., Cantarero I., Bellido M.L., Meyer S. (2017). Tetrahydrocannabinolic acid is a potent PPARgamma agonist with neuroprotective activity. Br. J. Pharmacol..

[34] Singh S.K., Antoine C., Tse C., Ji L., Reed M., Carter W.G., Trezza V., Bid H.K. (2026). Therapeutic potential of acidic cannabinoids: An update. J. Cannabis Res..

[35] Anderson L., Low I., Banister S., McGregor I. (2019). Pharmacokinetics of Phytocannabinoid Acids and Anticonvulsant Effect of Cannabidiolic Acid in a Mouse Model of Dravet Syndrome. J. Nat. Prod..

[36] Frautschy S.A., Baird A., Cole G.M. (1991). Effects of injected Alzheimer beta-amyloid cores in rat brain. Proc. Natl. Acad. Sci. USA.

[37] Kim J., Choi P., Park Y.T., Kim T., Ham J., Kim J.C. (2023). The Cannabinoids, CBDA and THCA, Rescue Memory Deficits and Reduce Amyloid-Beta and Tau Pathology in an Alzheimer’s Disease-like Mouse Model. Int. J. Mol. Sci..

[38] Viel T.A., Lima Caetano A., Nasello A.G., Lancelotti C.L., Nunes V.A., Araujo M.S., Buck H.S. (2008). Increases of kinin B1 and B2 receptors binding sites after brain infusion of amyloid-beta 1–40 peptide in rats. Neurobiol. Aging.

[39] Wakshlag J.J., Schwark W.S., Deabold K.A., Talsma B.N., Cital S., Lyubimov A., Iqbal A., Zakharov A. (2020). Pharmacokinetics of Cannabidiol, Cannabidiolic Acid, Delta9-Tetrahydrocannabinol, Tetrahydrocannabinolic Acid and Related Metabolites in Canine Serum After Dosing with Three Oral Forms of Hemp Extract. Front. Vet. Sci..

[40] Azzolini F., Gilio L., Pavone L., Iezzi E., Dolcetti E., Bruno A., Buttari F., Musella A., Mandolesi G., Guadalupi L. (2022). Neuroinflammation Is Associated with GFAP and sTREM2 Levels in Multiple Sclerosis. Biomolecules.

[41] Kumar V., Prabhu S.D., Bansal S.S. (2022). CD4(+) T-lymphocytes exhibit biphasic kinetics post-myocardial infarction. Front. Cardiovasc. Med..

[42] Nogai A., Siffrin V., Bonhagen K., Pfueller C.F., Hohnstein T., Volkmer-Engert R., Brück W., Stadelmann C., Kamradt T. (2005). Lipopolysaccharide injection induces relapses of experimental autoimmune encephalomyelitis in nontransgenic mice via bystander activation of autoreactive CD4+ cells. J. Immunol..

[43] Teixeira B., Bittencourt V.C., Ferreira T.B., Kasahara T.M., Barros P.O., Alvarenga R., Hygino J., Andrade R.M., Andrade A.F., Bento C.A. (2013). Low sensitivity to glucocorticoid inhibition of in vitro Th17-related cytokine production in multiple sclerosis patients is related to elevated plasma lipopolysaccharide levels. Clin. Immunol..

[44] De Petrocellis L., Ligresti A., Moriello A.S., Allarà M., Bisogno T., Petrosino S., Stott C.G., Di Marzo V. (2011). Effects of cannabinoids and cannabinoid-enriched Cannabis extracts on TRP channels and endocannabinoid metabolic enzymes. Br. J. Pharmacol..

[45] Zagzoog A., Mohamed K.A., Kim H.J.J., Kim E.D., Frank C.S., Black T., Jadhav P.D., Holbrook L.A., Laprairie R.B. (2020). In vitro and in vivo pharmacological activity of minor cannabinoids isolated from *Cannabis sativa*. Sci. Rep..

[46] Randjelovic P., Veljković S., Stojiljković N., Sokolovic D., Ilić I., Laketić D., Randjelović D., Randjelović N. (2015). The Beneficial Biological Properties of Salicylic Acid. Acta Fac. Medicae Naissensis.

[47] Liu J., Li H., Burstein S.H., Zurier R.B., Chen J.D. (2003). Activation and binding of peroxisome proliferator-activated receptor gamma by synthetic cannabinoid ajulemic acid. Mol. Pharmacol..

[48] Encinas J.M., Manganas L., Enikolopov G. (2005). Nitric oxide and multiple sclerosis. Curr. Neurol. Neurosci. Rep..

[49] Kim E.Y., Moudgil K.D. (2008). Regulation of autoimmune inflammation by pro-inflammatory cytokines. Immunol. Lett..

[50] Zakharova M., Ziegler H.K. (2005). Paradoxical anti-inflammatory actions of TNF-alpha: Inhibition of IL-12 and IL-23 via TNF receptor 1 in macrophages and dendritic cells. J. Immunol..

[51] Fresegna D., Bullitta S., Musella A., Rizzo F.R., De Vito F., Guadalupi L., Caioli S., Balletta S., Sanna K., Dolcetti E. (2020). Re-Examining the Role of TNF in MS Pathogenesis and Therapy. Cells.

[52] Batoulis H., Recks M.S., Holland F.O., Thomalla F., Williams R.O., Kuerten S. (2014). Blockade of tumor necrosis factor-alpha in experimental autoimmune encephalomyelitis reveals differential effects on the antigen-specific immune response and central nervous system histopathology. Clin. Exp. Immunol..

[53] Kassiotis G., Kollias G. (2001). Uncoupling the proinflammatory from the immunosuppressive properties of tumor necrosis factor (TNF) at the p55 TNF receptor level: Implications for pathogenesis and therapy of autoimmune demyelination. J. Exp. Med..

[54] Liu J., Marino M.W., Wong G., Grail D., Dunn A., Bettadapura J., Slavin A.J., Old L., Bernard C.C. (1998). TNF is a potent anti-inflammatory cytokine in autoimmune-mediated demyelination. Nat. Med..

[55] Estrada C., Gomez C., Martin C., Moncada S., Gonzalez C. (1992). Nitric oxide mediates tumor necrosis factor-alpha cytotoxicity in endothelial cells. Biochem. Biophys. Res. Commun..

[56] Aliyu M., Zohora F.T., Ceylan A., Hossain F., Yazdani R., Azizi G. (2024). Immunopathogenesis of multiple sclerosis: Molecular and cellular mechanisms and new immunotherapeutic approaches. Immunopharmacol. Immunotoxicol..

[57] Rock E.M., Limebeer C.L., Parker L.A. (2018). Effect of cannabidiolic acid and ∆ (9)-tetrahydrocannabinol on carrageenan-induced hyperalgesia and edema in a rodent model of inflammatory pain. Psychopharmacology.

[58] Goerl B., Watkins S., Metcalf C., Smith M., Beenhakker M. (2021). Cannabidiolic acid exhibits entourage-like improvements of anticonvulsant activity in an acute rat model of seizures. Epilepsy Res..

[59] Carmona-Hidalgo B., González-Mariscal I., García-Martín A., Prados M.E., Ruiz-Pino F., Appendino G., Tena-Sempere M., Muñoz E. (2021). Δ9-Tetrahydrocannabinolic Acid markedly alleviates liver fibrosis and inflammation in mice. Phytomedicine.

[60] Verhoeckx K.C., Korthout H.A., van Meeteren-Kreikamp A.P., Ehlert K.A., Wang M., van der Greef J., Rodenburg R.J., Witkamp R.F. (2006). Unheated Cannabis sativa extracts and its major compound THC-acid have potential immuno-modulating properties not mediated by CB1 and CB2 receptor coupled pathways. Int. Immunopharmacol..

[61] Fleisher-Berkovich S., Ventura Y., Amoyal M., Dahan A., Feinshtein V., Alfahel L., Israelson A., Bernstein N., Gorelick J., Ben-Shabat S. (2023). Therapeutic Potential of Phytocannabinoid Cannabigerol for Multiple Sclerosis: Modulation of Microglial Activation In Vitro and In Vivo. Biomolecules.

