# Supplementary material for: Anti-Neuroinflammatory Cannabinoid Acids as a New Therapeutic Approach for Multiple Sclerosis
Source: Molecules. 2026 Apr 7;31(7):1227. doi: 10.3390/molecules31071227 (PMC13074990; doi:10.3390/molecules31071227)
Supplement: Supplementary file 1 [file molecules-31-01227-s001.zip › molecules-4186531-supplementary.docx]

Supporting Information

Analytical validation of CBDA and THCA purity

# Overview

Liquid chromatography coupled to mass spectrometry (LC–MS) and UV detection was used to confirm the identity and purity of cannabidiolic acid (CBDA) and Δ⁹-tetrahydrocannabinolic acid (THCA) standards used in this study. Chromatographic analysis demonstrated high chemical purity for both compounds, with peak area normalization indicating purities ranging between **97–99%**.

Chromatographic retention times, UV absorbance spectra, and mass spectral characteristics were consistent with previously reported analytical data for CBDA and THCA, confirming the identity of the analyzed compounds and supporting their suitability for biological assays.

**Instrumentation and Chromatographic Conditions**

Chromatographic analyses were performed using a Waters Alliance HPLC system (Waters Corporation, Milford, MA, USA) equipped with an autosampler, quaternary solvent delivery module, column oven, and photodiode array (PDA) detector. Mass spectrometric detection was conducted using a Micromass Quattro Micro triple-quadrupole mass spectrometer (Waters/Micromass, Manchester, UK) with an electrospray ionization (ESI) source. Data acquisition and processing were performed using MassLynx software, and UV spectra were recorded over the range 200–400 nm.

Chromatographic separation was achieved on a reversed-phase C18 column (150 × 4.6 mm, 5 μm). The mobile phase consisted of water with 0.1% formic acid (A) and methanol with 0.1% formic acid (B). Gradient elution was used to achieve optimal separation of cannabinoid acids and minor impurities. Typical operating conditions included a flow rate of 0.5–1.0 mL min⁻¹, column temperature of ~30 °C, and an injection volume of 5–10 μL. Under these conditions, CBDA and THCA eluted as well-resolved peaks consistent with authentic cannabinoid standards.

.

**Mass Spectrometry and UV Spectral Confirmation**

Mass spectrometric detection was performed using electrospray ionization in positive ion mode (ESI⁺). Typical source parameters included a capillary voltage of ~3.0–3.5 kV, cone voltage of 25–40 V, source temperature of ~120 °C, and desolvation temperature of ~300–350 °C with nitrogen as the desolvation gas. Under these conditions, both CBDA and THCA generated prominent ions at m/z 341, consistent with characteristic in-source fragmentation of acidic cannabinoids.

UV spectra recorded with the PDA detector showed characteristic cannabinoid absorbance maxima near 210–230 nm, with a secondary band at ~270 nm, which is typical of aromatic carboxylic acid–containing cannabinoid structures. The combined agreement of retention time, UV spectra, and mass spectral features confirms the identity of CBDA and THCA in the analyzed samples.

# Chromatographic Analysis


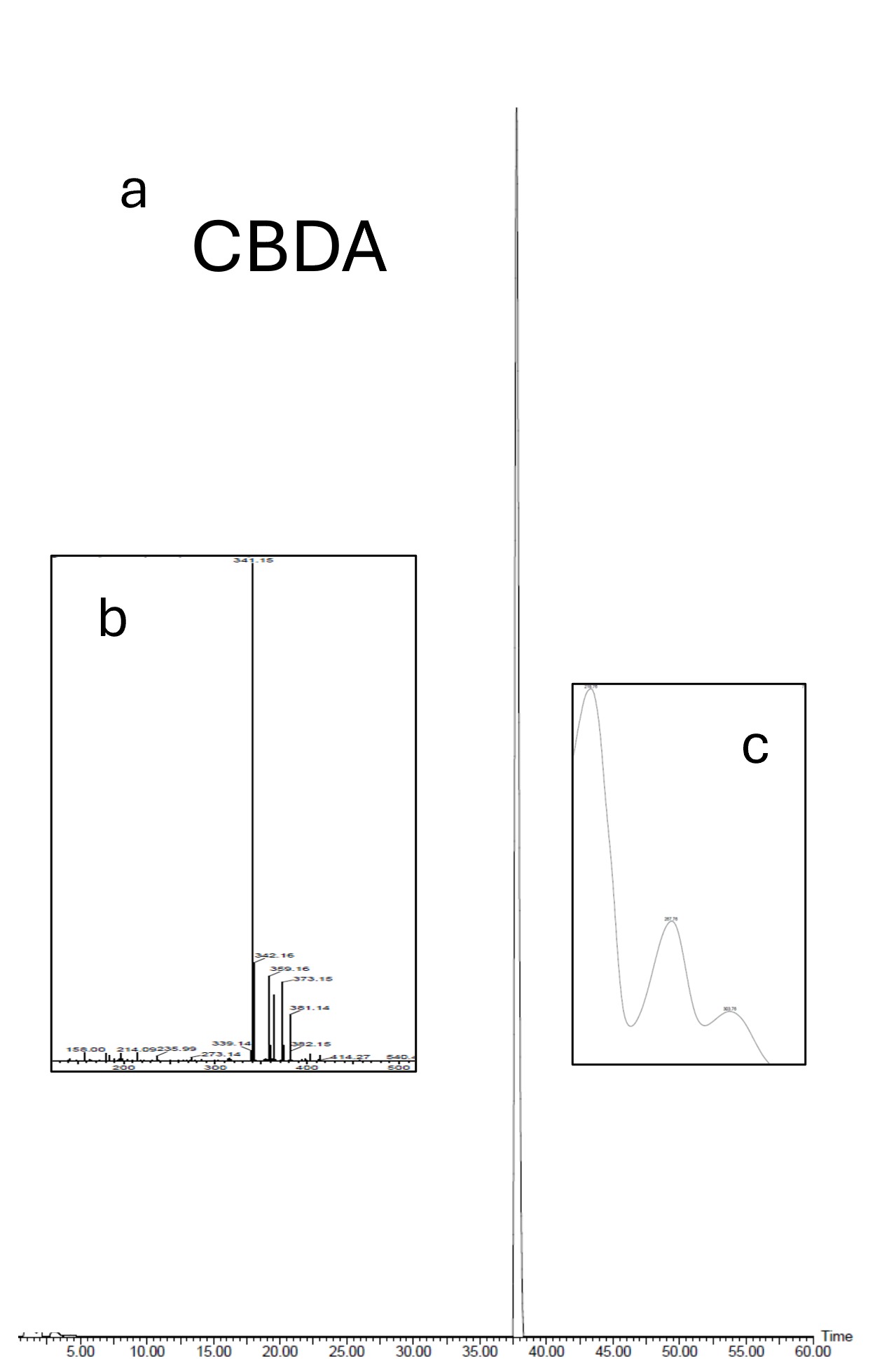


**Figure S1. Representative chromatographic and spectral characterization of CBDA.**

**(A)** HPLC chromatogram of the CBDA reference material recorded at 220 nm, showing a dominant peak corresponding to CBDA with only minor trace impurities. Peak area normalization indicated a purity of approximately **99%**.

**(B)** Mass spectrum obtained under ESI⁺ conditions for the CBDA chromatographic peak. The dominant ion observed at **m/z 341** is consistent with characteristic in-source fragmentation of acidic cannabinoids and matches reported LC–MS behavior for CBDA.

**(C)** UV absorbance spectrum extracted from the CBDA chromatographic peak. The spectrum shows characteristic cannabinoid absorbance maxima near **210–230 nm** and a secondary band at **~270 nm**, consistent with aromatic carboxylic acid–containing cannabinoid structures.


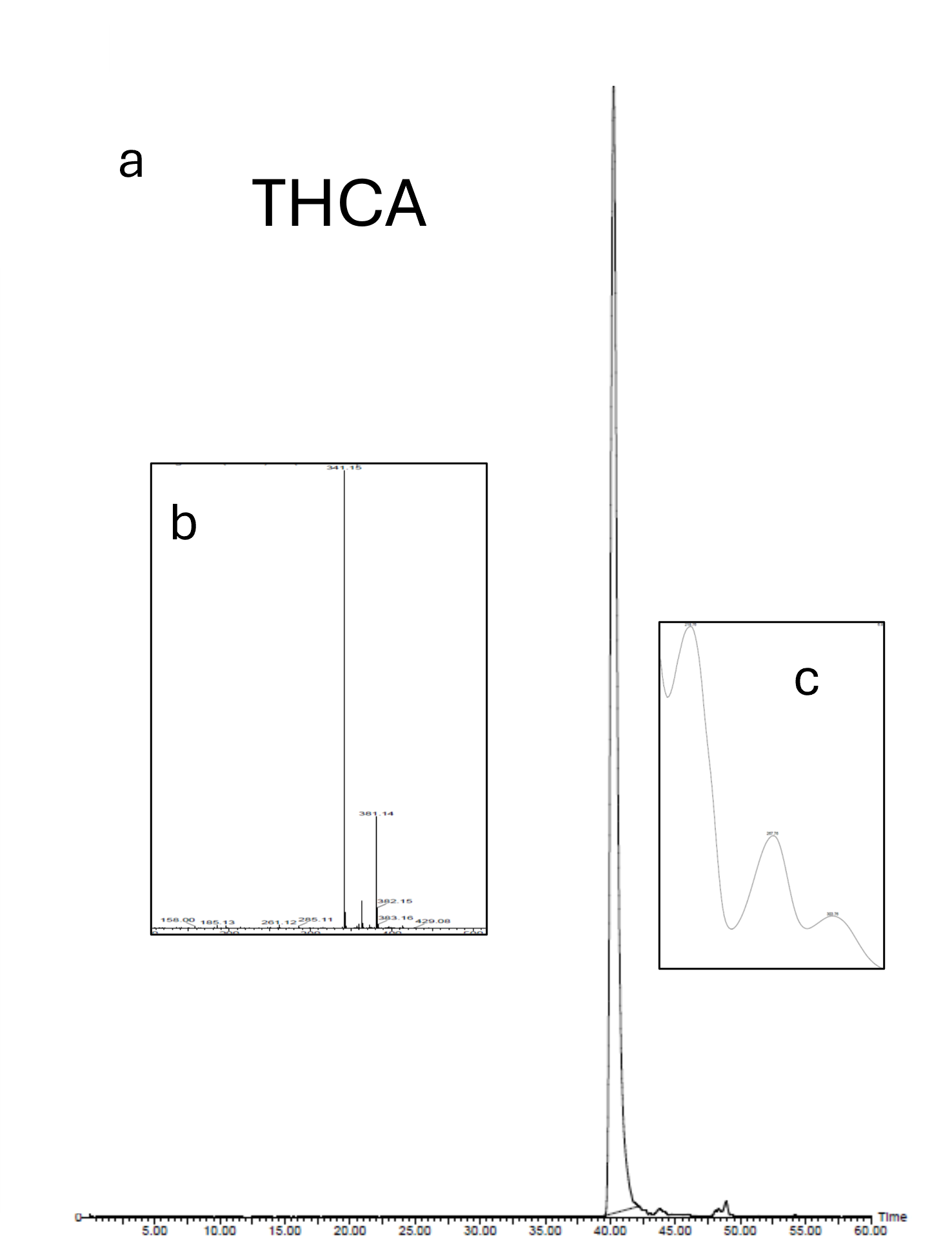


**Figure S2. Representative chromatographic and spectral characterization of THCA.**

**(A)** HPLC chromatogram of the THCA reference material recorded at 220 nm, showing a single dominant peak corresponding to THCA with minimal secondary peaks. Peak area normalization indicated a purity of approximately **97%**.

**(B)** Mass spectrum obtained under ESI⁺ conditions for the THCA chromatographic peak. A dominant ion at **m/z 341** was observed, consistent with expected fragmentation patterns of acidic cannabinoids under electrospray ionization.

UV absorbance spectrum extracted from the THCA chromatographic peak. The spectral profile shows the characteristic cannabinoid absorbance features near **210–230 nm** and **~270 nm**, consistent with previously reported spectra for THCA.

**(C)** UV absorbance spectrum extracted from the THCA chromatographic peak. The spectral profile shows the characteristic cannabinoid absorbance features near **210–230 nm** and **~270 nm**, consistent with previously reported spectra for THCA.
